# Phylogenetic groups and extraintestinal virulence genes of inflow Escherichia coli entering a municipal drinking water treatment facility (St. Paul, MN, USA)

**DOI:** 10.1099/mic.0.001542

**Published:** 2025-03-27

**Authors:** James R. Johnson, Brian D. Johnston, Paul Thuras

**Affiliations:** 1 Minneapolis VA Health Care System, Minneapolis, MN, 55417, USA; 2 Department of Medicine, University of Minnesota, Minneapolis, MN, 55455, USA; 3 Department of Psychiatry, University of Minnesota, Minneapolis, MN, 55454, USA

**Keywords:** drinking water, *Escherichia coli*, extraintestinal pathogenic *Escherichia coli *(ExPEC), phylogenetic group, public health, virulence gene

## Abstract

Extraintestinal pathogenic *Escherichia coli* (ExPEC), a leading cause of urinary tract infections, sepsis and neonatal meningitis, circulates between diverse hosts and the environment. Consequently, identifying ExPEC reservoirs and transmission pathways has potentially great public health importance. Here, we used PCR-based methods to characterize 104 *E. coli* isolates from inflow water to the St. Paul, MN (USA), municipal drinking water treatment plant. Isolates were analysed for major phylogenetic groups and multiple extraintestinal virulence genes. Additionally, from the 65 (of 104) water samples that yielded multiple *E. coli* colonies, we screened *E. coli* population DNA for virulence genes. Thirty-three percent of isolates represented virulence-associated groups B2 and D, and 8% (95% CI: 3%, 15%) qualified molecularly as ExPEC. The ExPEC isolates, all from group B2 or D, had a median virulence gene score of 11.0 and collectively contained all but four of the 28 studied extraintestinal virulence genes. Population DNA screening increased the proportion of samples positive for individual virulence genes and, presumptively, for ExPEC [14% (95% CI: 10%, 30%) vs. 8%, *P*=0.03]. These findings identify a previously underappreciated potential mechanism for community-wide dissemination of ExPEC and underscore the importance of consistent disinfection of municipal drinking water.

## Introduction

*Escherichia coli* is an important extraintestinal pathogen, with major health and economic consequences [1,2]. In humans, it is the leading cause of urinary tract infections, sepsis and neonatal meningitis and can cause infections in nearly all tissues and organs.

The distinctive *E. coli* strains that cause most extraintestinal *E. coli* infections – termed extraintestinal pathogenic *E. coli* (ExPEC) [3,4] – exhibit diverse combinations of specialized accessory traits that distinguish them from diarrhoeagenic *E. coli* and nonpathogenic commensal *E. coli* strains [4,5]. Such traits, often called extraintestinal virulence factors, variously facilitate attachment and colonization, e.g. P fimbriae (*pap*); tissue invasion, e.g. invasion of brain endothelium A (*ibeA*); tissue injury, e.g. cytotoxic necrotizing factor (*cnf1*); avoidance or subversion of host defences, e.g. group 2 capsules (*kpsM* II); iron acquisition, e.g. the aerobactin siderophore system (*iutA*); and diverse other fitness-enhancing functions. Unlike most diarrhoeagenic and nonpathogenic *E. coli*, ExPEC strains derive mainly from phylogenetic groups B2, D and F and from specific lineages (clones and sequence types) within the larger phylogenetic groups [4,6, 7].

In most extraintestinal *E. coli* infections, the causative strain derives immediately from the host’s own intestinal tract [8,10]. Indeed, ExPEC strains are excellent gut colonizers; they may persist in the gut for months or years without causing illness and may be passed amongst household members, including pets [11,13]. They may also be acquired from diverse non-human sources, including food-producing animals [14], retail foods [15], wild animals [16,17] and the external environment, including surface water [17].

Municipal drinking water in industrialized countries is commonly disinfected to prevent water-borne transmission of disease-causing micro-organisms, although with a focus mainly on diarrhoeagenic, not extraintestinal, pathogens [18]. The possibility that ExPEC also might be disseminated via the municipal drinking water system has been raised as a potential public health threat [19] but has been studied minimally, especially in industrialized countries. To date, potentially relevant studies addressed only intestinal pathogenic *E. coli*, were done in developing countries, assessed large watersheds or multiple surface water bodies without reference to a specific drinking water catchment, involved convenience samples of uncertain epidemiologic validity, assessed only finished water and/or included <30 isolates [20,27]. Here, we tested the hypothesis that inflow water to a municipal drinking water treatment facility in a high-income setting can contain ExPEC, which, in the absence of adequate disinfection, conceivably could be disseminated through the drinking water distribution system to community residents, potentially leading to extraintestinal infections. For this, we determined the presumptive ExPEC fraction amongst the facility’s inflow *E. coli* isolates and *E. coli*-containing water samples. To our knowledge, this study provides the first reported phylotyping and extraintestinal virulence genotyping of *E. coli* isolates from input water to a municipal drinking water treatment facility in an industrialized country.

## Methods

### Water samples: sources, collection and processing

The Saint Paul Regional Water Service (SPRWS) provides drinking water to the city of St. Paul, MN, from its drinking water treatment plant. The plant’s inflow water derives mainly from the Mississippi River. Water is pumped from the river through a long conduit to a chain of lakes upstream of the plant, the last of which is Lake Vadnais (in an adjacent suburb), from where the water is pumped through another conduit to the treatment plant. Water from a separate chain of lakes also flows into Lake Vadnais, thereby providing a minor additional input to the treatment plant. [This second chain of lakes, which historically was heavily impacted by surface runoff from the surrounding area and tended to have high bioburdens, has undergone extensive bioremediation efforts since the study period, resulting in reduced bioburdens (personal communication, Alexis Rossow, SPRWS).] In the treatment plant, after flocculation to remove solids and some micro-organisms, water hardness is adjusted, and chlorine and ammonia are added for disinfection.

For continuous microbiological monitoring, SPRWS systematically collects surveillance samples of input drinking water at multiple locations along the inflow water pathway. Samples are collected using a dipper connected to a rope, then filling 100 ml laboratory-prepared sterilized Nalgene sample containers. Locations sampled included the intake point of the Mississippi River conduit, sites along both chains of feeder lakes, Lake Vadnais, the terminal conduit and the treatment plant’s flocculation chamber. In 2001–2002, the plant’s laboratory filtered a portion of each water sample (from 1 to 100 ml, depending on the sample’s gross appearance) through a sterile 0.45 mm filter, which was then placed on an Membrane Fecal Coliform agar plate [28,29]. Plates were incubated for 24 h at 45.5 (+/−0.5) ^0^C to isolate, presumptively identify and quantify faecal coliforms and *E. coli*. Dark blue colonies were regarded presumptively as *E. coli*. From September 2001 through June 2002, culture plates that yielded presumptive *E. coli* were transferred to the research laboratory for further testing. Data regarding the total number of samples collected, their distribution by date and location and the proportion that yielded *E. coli* were not provided.

SPRWS also systematically collects and cultures post-treatment drinking water samples, both at the plant and from multiple points throughout the distribution system. These cultures are uniformly negative for *E. coli* or, if positive, prove to be falsely so, e.g. from faulty collection (personal communication, Alexis Rossow, SPRWS).

### Characterization of *E. coli* isolates

Upon receipt in the research laboratory, one presumptive *E. coli* colony per *E. coli*-positive input drinking water sample underwent identity confirmation based on citrate utilization, indole production and colonial appearance on eosin-methylene blue agar. Citrate-negative, indole-positive colonies with a characteristic *E. coli* appearance (green, with metallic sheen) were regarded as *E. coli*. Each primary culture plate’s arbitrarily selected index *E. coli* isolate, plus, for plates with ≥2 colonies, a population sample (i.e. plate sweep) containing all bacterial growth on the plate (which presumably included all *E. coli* genomes present), was frozen in 20% glycerol in broth at −80 °C for subsequent analysis.

PCR testing was done upon completion of sample accrual. For this, broth cultures were prepared from frozen stocks of index isolates and, for samples with ≥2 *E*. *coli* colonies, of population growth. After overnight incubation at 37 °C, the turbid broth was centrifuged, and the supernatant was used as target DNA. Testing was done in duplicate for each DNA preparation, using for each PCR target appropriate positive and negative control strains, as defined based on previous genotyping [30]. Established endpoint PCR-based assays were used to determine the major *E. coli* phylogenetic group (index isolates only; groups F and G were not resolved separately from groups B2 and D by the assay used) [31] and, using PCR conditions and primers as described elsewhere, to screen for eight key extraintestinal virulence genes (index isolates and population samples) [30,32].

According to an established molecular definition, isolates positive for ≥2 of five *E. coli*-specific ExPEC indicator genes – *papAH* and/or *papC* (P fimbriae; counted as one), *sfa/focDE* (S and F1C fimbriae), *afa/draBC* (Dr-binding adhesins), *kpsM* II (group 2 capsules) and *iutA* (aerobactin system) – were regarded as ExPEC-positive [33]. (The same definition was used with population DNA samples, but because of the possibility of summing across multiple different genomes in a population, including possibly non-*E. coli*, population samples that fulfilled the criteria for ExPEC were regarded only as potentially ExPEC-positive.) Using established PCR conditions and primers, ExPEC-positive isolates (but not ExPEC-positive mixed-growth samples) were tested for 20 additional extraintestinal virulence genes, giving 28 genes total [30,34]. They also were tested for 3 alleles of *papG* (P fimbriae adhesin molecule) [35] and 11 F antigen-encoding alleles of *papA* (P fimbriae structural subunit) [36]. An isolate’s virulence gene score was the total number of virulence genes detected, adjusted for multiple detections of certain operons.

### Statistical analysis

Specimen collection and molecular testing were completed by 2008. Data analysis was delayed until 2023 by logistical considerations. For analysis purposes, water samples were regarded as positive for a particular *E. coli* genotype based on two different definitions: presence of the gene in the sample’s index *E. coli* isolate (restrictive definition) or, alternatively, in the sample’s index isolate and/or its mixed-growth population, if any (inclusive definition). Comparisons of proportions were tested using a chi-squared test or Fisher’s exact test for unpaired comparisons and McNemar’s test for paired comparisons. Throughout, the criterion for statistical significance was *P*<0.05 (two-tailed). Given the exploratory, hypothesis-generating nature of the study, no adjustment was made for multiple comparisons, to avoid possible type-2 errors from being overly stringent [37].

## Results

### Study population

During the 10-month study period (September 2001–June 2002), the SPRWS recovered *E. coli* from 104 routine surveillance cultures of surface water collected from diverse sites upstream from, or early within, the St. Paul, MN, municipal drinking water treatment facility. Culture-positive samples were from two sites along the feeder lake chain leading from the Mississippi River (*n*=51 samples); four sites along the other feeder lake chain (*n*=4); Lake Vadnais, into which the feeder lake chains empty (*n*=41); the terminal conduit, which carries water from Lake Vadnais into the treatment facility (*n*=4); and the flocculation chamber (*n*=4). Of the 104 *E. coli*-positive water samples, 39 yielded a single *E. coli* colony, and 65 yielded ≥2 colonies (range, 2–140). With cultured water volumes (which varied depending on the sample’s visual appearance) ranging from 1 to 100 ml (median, 2 ml), the corresponding estimated *E. coli* concentrations ranged from 0.02 to 15 c.f.u. ml^−1^ (median, 1.0 c.f.u. ml^−1^) for the 99 samples with concentrations that fell within the quantifiable range, whereas five samples had concentrations exceeding the quantification limit. One colony per sample was selected arbitrarily as the sample’s index isolate.

### Phylogenetic group

PCR-based determination of the major *E. coli* phylogenetic group for the 104 index isolates showed a predominance of (presumptive) group A (52%), with the remaining isolates divided roughly evenly between groups B1, B2 and D (Table 1). Index isolates from the 65 samples (i.e. primary plates) that yielded ≥2 colonies exhibited a similar phylogenetic distribution to the total index isolate population, consistent with their representing the same source population (Table 1).

**Table 1.** Phylogenetic groups and screening virulence genes amongst index *E. coli* isolates and mixed DNA from inflow water to a municipal drinking water treatment plant

	All samples: no. of positive (% of 104)		Samples with ≥2 colonies: no. of positive (% of 65)		
Variable*	Index isolate	Index† and/or mixed DNA	Index isolate	Index† and/or mixed DNA	*P* value‡, index vs. index and/or mixed DNA
Group A	54 (52)	na§	33 (51)	na§	na§
Group B1	16 (15)	na§	9 (14)	na§	na§
Group B2	20 (19)	na§	13 (20)	na§	na§
Group D	14 (14)	na§	10 (15)	na§	na§
*papAH*	6 (6)	11 (11)	4 (6)	9 (14)	0.06
*papC*	6 (6)	10 (10)	4 (6)	8 (12)	0.13
*sfa/focDE*	4 (4)	8 (8)	2 (3)	6 (9)	0.13
*afa/draBC*	1 (1)	1 (1)	1 (1.5)	1 (1.5)	1.0
*iutA*	3 (3)	5 (5)	3 (5)	5 (8)	0.50
*kpsM* II	19 (18)	32 (31)	15 (23)	28 (43)	<0.001
*hlyD*	4 (4)	8 (8)	2 (3)	6 (9)	0.13
*fimH*	89 (86)	98 (94)	55 (85)	64 (98)	0.004
ExPEC	8 (8)	14 (14)	6 (9)	12 (18)	0.03

* Definitions: *papAH*, P fimbriae structural subunit; *papC*, P fimbriae assembly; *sfa/focDE*, S and F1-C fimbriae; *afa/draBC*, Dr-binding adhesins; *iutA*, aerobactin receptor; *kpsM* II, group 2 capsules; *hlyD*, alpha haemolysin; *fimH*, type-1 fimbriae adhesin; ExPEC is defined operationally as the presence of ≥2 of *papAH* and/or *papC*, *sfafocDE*, *afa/draBC*, *iutA* and *kpsM* II.

† Index isolate or mixed (i.e. population) DNA.

‡ *P* values (by McNemar’s test, two-tailed) are for comparisons of results for index isolates only (restrictive definition) vs. index isolate and/or mixed (i.e. population) DNA (inclusive definition). *P* values were identical whether the comparisons included all 104 samples or only the 65 that yielded ≥2 *E*. *coli* colonies.

§ na, not applicable. The phylogenetic group cannot be determined for mixed (population) samples by using the cited Clermont PCR assay.

### Screening virulence genotypes

All eight screening ExPEC virulence genes were detected in at least one index isolate each, at frequencies ranging from 1% [*afa/draBC* (Dr-binding adhesins)] to 86% [*fimH* (type-1 fimbriae adhesin)] (Table 1). The second-most-prevalent screening virulence gene was *kpsM* II (group 2 capsules) (18%), whereas the remaining genes had <10% prevalence each. Eight (8%; 95% CI: 3%, 15%) isolates qualified molecularly as ExPEC by virtue of containing ≥2 of the five ExPEC-defining virulence genes, i.e. *papA* (P fimbriae structural subunit) and/or *papC* (P fimbriae assembly), *sfa/focDE* (S and F1C fimbriae), *afa/draBC*, *kpsM* II (group 2 capsules) and *iutA* (aerobactin receptor) [33]. The index isolates from the 65 samples (i.e. primary plates) that yielded ≥2 *E*. *coli* colonies exhibited a similar frequency of screening virulence genes and ExPEC status as did the 104 total index isolates (Table 1), again consistent with their representing the same source population as the 39 index isolates from single-colony samples.

Amongst the 104 index isolates, six of the eight screening virulence genes and ExPEC status were distributed significantly by phylogenetic group, being most abundant in group B2, followed by group D (Table 2). The only exception to this pattern involved *afa/draBC*, which was detected only in a single group D isolate.

**Table 2.** Phylogenetic group distribution of screening virulence genotypes amongst 104 index *E. coli* isolates from inflow water to a municipal drinking water treatment plant

	No. of isolates with genotype (column %)					
Variable*	Total(*n*=104)	Group A†(*n*=54)	Group B1† (*n*=16)	Group B2† (*n*=20)	Group D† (*n*=14)	Four-group *P* value‡
*papAH/C*	6 (6)	0 (0)	0 (0)	6 (30)	0 (0)	<0.001
*sfa/focDE*	4 (4)	0 (0)	0 (0)	4 (20)	0 (0)	0.001
*afa/draBC*	1 (1)	0 (0)	0 (0)	0 (0)	1 (7)	0.09
*iutA*	3 (3)	0 (0)	0 (0)	2 (10)	1 (7)	0.09
*kpsM* II	19 (18)	6 (11)	1 (6)	9 (45)	3 (21)	0.004
*hlyD*	4 (4)	0 (0)	0 (0)	4 (20)	0 (0)	0.001
*fimH*	89 (86)	39 (72)	16 (100)	20 (100)	4 (100)	<0.001
ExPEC	8 (8)	0 (0)	0 (0)	7 (35)	1 (7)	<0.001

* Definitions: *papAH*, P fimbriae structural subunit; *papC*, P fimbriae assembly (all isolates with *papA* had *papC*, and vice versa); *sfa/focDE*, S and F1-C fimbriae; *afa/draBC*, Dr-binding adhesins; *iutA*, aerobactin receptor; *kpsM* II, group two capsules; *hlyD*, alpha haemolysin; *fimH*, type-1 fimbriae adhesin; ExPEC is defined operationally as the presence of ≥2 of *papAH* and/or *papC*, *sfafocDE*, *afa/draBC*, *iutA* and *kpsM* II.

† Phylogenetic groups A, B1, B2 and D accounted for all 104 index isolates.

‡ Four-group comparison *P* values, as calculated using a two-tailed chi-squared test.

PCR-based testing of population DNA for the 65 water samples that yielded ≥2 *E*. *coli* colonies resulted in additional detection of virulence genes, presumably due to the presence in certain mixed samples of one or more minor (non-index) strains carrying the gene of interest (Table 1). Overall, the incremental yield from population DNA screening was proportionately greatest – amounting to approximately a doubling of the estimated frequency – for *papAH*, *papC*, *sfa/focDE* and *iutA* (Table 1). This effect, although inherently limited to the subset of samples that yielded multiple colonies, nonetheless was evident in the overall population, despite dilution by the single-colony samples. Using an inclusive definition, i.e. detection of a gene in the sample’s index isolate and/or population DNA, 14% of all samples (95% CI: 8%, 23%) – and 18% of the 65 samples with ≥2 *E*. *coli* colonies (95% CI: 10%, 30%) – potentially contained ExPEC, assuming that a single strain contained all the ExPEC-defining genes detected in a population DNA sample.

### Characteristics of ExPEC isolates

The eight ExPEC index isolates were all derived from phylogroup B2 (*n*=7) or D (*n*=1). Thus, within the index isolate population, the ExPEC fraction was significantly higher within groups B2 and D combined (8 out of 34, 24%) than within groups A and B1 combined (0 out of 70, 0%) (*P*<0.001, Fisher’s exact test). Extended virulence genotyping of the eight ExPEC isolates detected, at varying frequencies, 24 of the 28 target virulence genes (including representatives of all functional categories studied), plus six of the 11 *papA* alleles and all 3 main *papG* alleles (Table 3). Aggregate virulence gene scores (median, 11.0; range, 7.0–11.75) were higher for the seven group B2 ExPEC isolates (median, 11.0; range, 7.5–11.75) than the single group D ExPEC isolate (7.0).

**Table 3.** Source details and bacterial characteristics of eight index ExPEC* isolates from inflow water to a municipal drinking water treatment plant

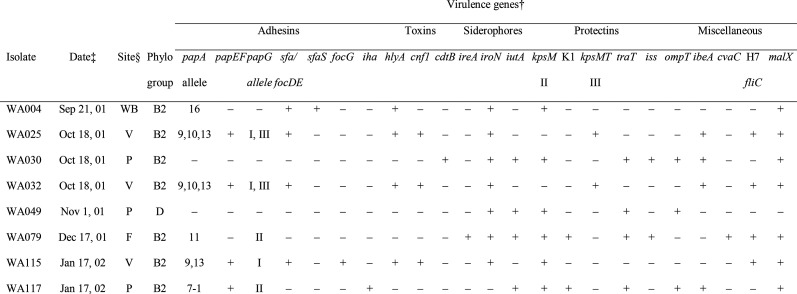 ­

*ExPEC, extraintestinal pathogenic *Escherichia coli*.

†Plus sign, gene detected. Minus sign, gene not detected. Virulence gene definitions: *papAH/C/EF/G*, P fimbriae structural subunit, assembly, minor pilins, and adhesin; *sfa/focDE*, S and F1-C fimbriae; *sfaS*, S fimbriae adhesin; *focG*, F1-C fimbriae adhesin; *iha*, adhesin-siderophore; *hlyA*, alpha hemolysin; *cnf1*, cytotoxic necrotizing factor 1; *cdtB*, cytolethal distending toxin; *ireA*, catecholate siderophore; *iroN*, salmochelin receptor; *iutA*, aerobactin receptor; *kpsM* II, group 2 capsules; K1, group 2 capsule variant; *kpsMT* III, group 3 capsules; *traT*, serum resistance-associated; *iss*, increased serum survival; *ompT*, serum resistance-associated outer membrane protein T; *ibeA*, invasion of brain endothelium; *cvaC*, colicin (microcin) V; H7 *fliC*, flagellar variant; *malX*, pathogenicity island marker. Listed genes were detected in 1-7 isolates each. All isolates had *fimH* (type-1 fimbriae adhesin) and *fyuA* (yersiniabactin receptor)*.* All isolates with *papAH* (P fimbriae structural subunit, represented here by the *papA* alleles) also had *papC*. Genes sought but not detected: *afa/draBC* (Dr-binding adhesins), *bmaE* (M fimbriae), *gafD* (G fimbriae), *kpsM* K2 (group 2 capsule variant).

‡Date of specimen collection.

§Specimen collection site codes: F, flocculation chamber (within water purification plant); P, Lake Pleasant (part of the first chain of lakes); V, Lake Vadnais; WB, White Bear storm sewer (part of the second chain of lakes).

## Discussion

In this study, we characterized *E. coli* from 104 geographically and temporally distributed samples of inflow water to a municipal drinking water treatment plant. Individual *E. coli* isolates were analysed for phylogenetic group and multiple extraintestinal virulence genes, and population DNA from the subset of samples that yielded multiple *E. coli* colonies was screened for virulence genes. We found that although most isolates appeared innocuous with respect to extraintestinal infection potential, a minority represented ExPEC and, hence, possibly could pose a human health threat if not removed by the treatment process. We also found that population DNA screening was more sensitive than single-isolate screening for identifying presumptively ExPEC-containing water samples. These findings document a previously neglected benefit of municipal drinking water supply disinfection, i.e. removal of ExPEC, and suggest that lapses in disinfection could have an added adverse human health consequence by allowing community-wide, water-borne dissemination of ExPEC, thereby facilitating the occurrence of extraintestinal *E. coli* infections [19].

Most isolates exhibited few or no extraintestinal virulence genes and presumptively were from phylogenetic groups that classically are associated with low extraintestinal virulence (groups A and B1) [38,39]. Together with the treatment plant’s consistently negative post-treatment surveillance cultures (personal communication, Alexis Rossow, SPRWS), this provides reassurance that, at least in the study locale at the time of the study, the municipal drinking water supply is an extremely unlikely source for ExPEC exposure amongst community dwellers.

Nonetheless, an appreciable minority of isolates, and an even greater proportion of water samples (according to population DNA screening), represented or presumptively contained ExPEC [33]. Moreover, the distinctive virulence gene profiles of several of the group B2 ExPEC isolates suggested membership in classic disseminated ExPEC lineages that commonly cause infections in humans. For example, isolates WA025, WA032 and WA115 had *papA* alleles F9 and F13 (+/−F10), *papG* allele I (+/−III) and *sfa/focDE*, *hlyA* and *cnf*, consistent with membership in the ‘J96-like’ clonal group within ST12 [40,41]. Likewise, isolate WA079 had *papA* F11, *papG* allele II, the K1 *kpsMT* II allele, *iutA*, *cvaC* and *fliC* H7, consistent with membership in the meningitis and cystitis-associated O1/O2:K1:H7 clonal group within ST95 [7,42, 43].

The presence of such strains in a drinking water treatment facility’s input water indicates that even a transient lapse in disinfection efficacy conceivably could result in a bolus of ExPEC strains of potential human health relevance passing through the distribution system, adding to the recognized public health threat of drinking water-borne diarrhoeagenic organisms [18]. This aligns with the findings of what, to our knowledge, was the only previous assessment of the extraintestinal virulence characteristics of municipal drinking water-source *E. coli* in an industrialized setting [20]. There, two of 28 post-treatment *E. coli* drinking water isolates qualified molecularly as ExPEC. This suggests that consistently effective municipal drinking water disinfection is an important safeguard against water-borne dissemination of ExPEC, as well as of diarrhoeagenic pathogens [19].

The observed associations of virulence genes and ExPEC status with phylogenetic groups B2 and D, and the specific virulence genes identified, correspond with familiar patterns from other contexts and locales [38,44, 45]. Thus, the *E. coli* population in inflow drinking water most likely derives from the larger general *E. coli* population, with possible inputs from diverse mammalian, avian or reptilian sources. That scenario contrasts with the evidence that some surface water reservoirs contain self-sustaining, free-living *E. coli* that exist independently of inputs from vertebrate hosts [46].

The enhanced detection of presumptive ExPEC by screening population DNA, in addition to single isolates, suggests that the water-borne *E. coli* population is clonally diverse. Although this might seem predictable, it is conceivable that the local *E. coli* population at a particular sampling site could be monoclonal, for example, due to the local dominance, or a passing bolus, of a particular clone. Here, clonal diversity was documented empirically for those samples that yielded multiple *E. coli* colonies and, by extension, likely is true also for at least some of those samples that yielded only a single colony, given the insensitivity of the sampling method used for detecting low *E. coli* concentrations. Indeed, it seems probable that more water samples would have been *E. coli*-positive had larger volumes been cultured or had broth amplification been used.

The health risk from ingesting a low dose of ExPEC, whether from drinking water or any other source, is not known. For most diarrhoeagenic *E. coli*, which, in industrialized countries, tend not to establish long-term human gut colonization, a comparatively large ingested dose is needed for the disease to occur, and if the disease does occur, it happens shortly after exposure [47,48]. By contrast, ExPEC are excellent gut colonizers and can amplify and persist in the host for months or years after acquisition, possibly leading to seemingly ‘sporadic’ disease cases in stably colonized hosts, long after a remote point-source dissemination event [11,49,52].

Although the effective colonizing dose for ExPEC is undefined, even a single ingested organism conceivably may suffice, especially for hosts with reduced gastric acidity, which is now common due to the widespread use of acid-blocking drugs [53]. The transmission of intestinal ExPEC strains amongst asymptomatic household members suggests that even minuscule ingested doses of ExPEC may suffice for establishing colonization [12,49, 54]. Thus, pending further study of this question – which may warrant a quantitative risk assessment – even low concentrations of ExPEC in drinking water must be regarded as a potentially significant health threat. Given the likely impossibility of managing inflow catchments so as to maintain sterility or absence of ExPEC, the possible threat posed by any ExPEC in drinking water suggests that consistently effective drinking water disinfection is an important safeguard against water-borne ExPEC dissemination.

The study’s main limitation is the remoteness of sample collection, which creates uncertainty regarding the study findings’ applicability to the current situation at this particular facility. However, the point of the study was mainly proof of concept, i.e. to determine whether a municipal drinking water system in an industrialized setting, using modern treatment methods, conceivably could be a source for dissemination of ExPEC within a community, and not specifically to document the studied system’s current status. As such, the possibility of shifts over time in the microbiological characteristics of input water to this particular treatment facility is less important than the demonstration that, indeed, ExPEC can be present in such a facility’s inflow water. The generalizability of this finding to other drinking water treatment facilities, locales and time points is indeterminate; new studies are needed to determine this. Nonetheless, this possibility of community-wide dissemination of water-borne ExPEC that this finding raises provides a cautionary note regarding another class of pathogens that such facilities must remove from drinking water.

The study has other limitations. It was based at a single facility, presumed for some analyses that multiple virulence genes detected in a population DNA sample may have all derived from the same strain (potentially falsely identifying ExPEC), did not assess for transmission, used an older version of the PCR phylotyping assay that misclassifies some non-group A strains as group A, did not adjust for multiple comparisons (making the findings more suggestive than definitive) and provided limited characterization of the *E. coli* isolates, including for genetic features that could be revealed by, for example, genome sequencing. It also did not assess for potentially harmful water-borne genetic elements – e.g. virulence and antimicrobial resistance genes – other than those within viable *E. coli* cells, detection of which would require molecular testing of water samples. It nonetheless has notable strengths, including the comparatively large overall sample size, the novel testing of input *E. coli* to a municipal drinking water facility in an industrialized country for phylogenetic groups and diverse extraintestinal virulence genes and the comparison of single-colony versus population DNA screening in this context.

## Conclusions

In summary, we presumptively detected ExPEC in 8%–14% of *E. coli*-positive surveillance samples of inflow water to a municipal drinking water treatment facility. The ExPEC isolates were from phylogenetic groups B2 and D and exhibited extensive virulence gene profiles, some suggesting known ExPEC lineages of human health importance. The likelihood of presumptively detecting ExPEC in an *E. coli*-positive sample varied with the sample’s *E. coli* concentration, implying polyclonality. These findings provide what, to our knowledge, is the first reported phylotyping and extraintestinal virulence genotyping of *E. coli* isolates from input water to a municipal drinking water treatment facility in an industrialized country. They suggest that disinfection of municipal drinking water may provide a previously under-appreciated added public health benefit by reducing the likelihood of disseminating ExPEC through the drinking water distribution system.

## Abbreviations

ExPEC: extraintestinal pathogenic *Escherichia coli*
SPRWS: Saint Paul Regional Water Service
